# Antiviral treatment of COVID-19: which role can clinical parameters play in therapy evaluation?

**DOI:** 10.1007/s15010-023-02081-0

**Published:** 2023-08-09

**Authors:** Yannis R. Hübner, Nikolai Spuck, Moritz Berger, Stefan Schlabe, Gereon J. Rieke, Sven Breitschwerdt, Kathrin van Bremen, Christian P. Strassburg, Maria A. Gonzalez-Carmona, Jan-Christian Wasmuth, Jürgen K. Rockstroh, Christoph Boesecke, Malte B. Monin

**Affiliations:** 1 Department of Internal Medicine I, University Hospital Bonn, Venusberg-Campus 1, 53127 Bonn, Germany; 2 Faculty of Medicine, Institute of Medical Biometry, Informatics and Epidemiology, University of Bonn, Bonn, Germany; 3 German Centre for Infection Research (DZIF), partner-site Cologne-Bonn, Bonn, Germany

Dear Editor,

With great interest, we read the article “Use and Effectiveness of Remdesivir for the Treatment of Patients with COVID-19 Using Data from the Lean European Open Survey on SARS-CoV-2 infected patients (LEOSS): A multicentre cohort study”, published in Infection by Pilgram et al. [1]. According to the data, initiation of treatment with remdesivir in the advanced course of COVID-19 was effective.

Our own data basically confirm the results and add further insights. In summary, we do propose to use a simple score based on clinical data, both, for therapy decision-making and for therapy evaluation.

The COVID-19 pandemic has created a global public health crisis with the need for treatment options to alleviate symptoms and prevent spreading of the virus. Direct acting antivirals (DAA) and monoclonal antibodies (mABs) neutralizing SARS-CoV-2 are recommended for treatment by the World Health Organization (WHO) based on pivotal studies and real-world data mainly focusing on virological parameters [2]. In contrast, more robust clinical data as presented by Pilgram et al. are still limited [1]. Of note, for the time being, mABs are only effective to a very limited extent due to viral evolution and their use is therefore reserved for individual cases. The focus was therefore placed on the data on the use of RDV.

We here mapped clinical outcomes using the WHO ordinal clinical severity scale (hereafter: WHO ordinal scale) to assess the effectiveness of remdesivir (RDV) and the mAB combination casirivimab/imdevimab (CVIV).

This retrospective, single-centre cohort study aimed to assess the effect of treatment of COVID-19 on the clinical course of patients admitted to the infectious disease department of the University Hospital Bonn, Germany, between March 2020 and November 2021.

The diagnosis of COVID-19 had to be PCR-confirmed. Patients were assigned to two groups based on the treatment they received during their hospital stay: the RDV group and the CVIV group.

RDV therapy was initiated immediately in patients requiring oxygen (the latest with an oxygen saturation of 90.0%). Median time from diagnosis to initiation of RDV therapy was 7 days (IQR 4–10 days). Vaccinated patients and patients receiving a combination of RDV and CVIV and/or dexamethasone were excluded. Therapeutic effect was assessed comparing unvaccinated patients having received RDV to unvaccinated, untreated control patients requiring oxygen support.

The CVIV group included patients who received this mAB within the first five days after onset of the infection. The control group comprised patients hospitalized within the first five days after onset of the infection not treated with CVIV. For those groups, vaccination and combination of CVIV and RDV and/or dexamethasone in the further course were no exclusion criteria.

The clinical course was assessed using the WHO ordinal scale. Each patient was scored with zero to eight points: 0: no clinical or virological evidence of infection; 1: ambulatory, no activity limitation; 2: ambulatory, activity limitation; 3: hospitalized, no oxygen therapy; 4: hospitalized, oxygen mask or nasal prongs; 5: hospitalized, noninvasive mechanical ventilation or high-flow nasal cannula; 6: hospitalized, intubation and invasive mechanical ventilation (IMV); 7: hospitalized, IMV + additional support such as pressors or extracardiac membranous oxygenation; 8: death [3]. The primary endpoint in the RDV group was the WHO ordinal scale score of patients requiring oxygen support (WHO ordinal scale score > 3) treated with RDV compared to untreated patients with WHO ordinal scale score > 3. To assess the efficacy of CVIV, it was analysed whether oxygen therapy was necessary under CVIV (WHO ordinal scale score > 3) compared to a control group not treated with CVIV. In both groups, the worst WHO ordinal scale score was determined for each patient for the comparative analyses. The effects of RDV on length of hospitalization and duration of positive PCR results were secondary endpoints.

Raw data were collected and organized in an electronic case report form. All data were de-identified and kept confidential to maintain patients’ privacy. Statistical analysis was performed using R software (version 4.0.3). Inverse probability of treatment weights were determined using propensity scores in order to account for possibly unequal distributions of important risk factors between the treatment groups. That is, observations in the treatment group that are similar to the expected observation in the control group are weighted more strongly, and vice versa. Propensity scores were calculated based on the most frequently documented risk factors, i.e., age over 65 years, hypertension, obesity, diabetes, chronic kidney disease, heart disease, chronic obstructive pulmonary disease, and neurological disease, as defined by the Center for Disease Control and Prevention (CDC) [4], and the total number of present risk factors. Vaccination status was used as an additional factor in the CVIV analysis. We applied inverse probability weighting as it allows the use of the complete sample in the analysis and facilitates the estimation of the average treatment effect, whereas, when propensity score matching is used, patients for whom no suitable match is available may be excluded and only the average effect on the treated is estimated [6]. An ordered logistic regression model was applied to the weighted data to analyse the effect of RDV on the WHO ordinal scale. Cumulative incidence functions were calculated to assess the effects on the length of hospitalization and duration of positive PCR results. Confidence intervals (CI) were constructed based on 500 bootstrap samples. The effect of CVIV on need for oxygen support (WHO ordinal scale score > 3) was investigated using a binary logistic model. The study was conducted in accordance with the Declaration of Helsinki.

A total of 394 patients who were diagnosed with PCR-confirmed COVID-19 were included in this study, with a mean age of 60.3 ± 18 years. As shown in Table 1, age greater than 65 years was identified as the most frequently observed risk factor in the data. Of all patients, 10.9% (n = 43/392) received oxygen therapy and were treated with remdesivir (RDV) alone, while 19.3% (n = 76/392) were hospitalized within five days after infection and received CVIV treatment. 6.3% (n = 25/394) were treated with CVIV and, in the further course, additionally with RDV. The control groups included 12.4% (n = 49/394) and 41.2% (n = 162/394) of the patients, respectively. 16.2% (n = 64/394) were excluded due to the criteria mentioned above. The detailed distribution of risk factors for severe COVID-19 in the study population is shown in Table 1.

**Table 1:** Baseline characteristics of this studies population

	RDV analysis data		CVIV analysis data		Overall (N = 394)
	No RDV (N = 49)	RDV (N = 43)	No CVIV (N = 162)	CVIV (N = 76)	
Gender (male)	20 (40.8%)	25 (58.1%)	84 (51.9%)	42 (55.3%)	212 (53.8%)
Age in years, mean ± SD	66.5 ± 19.2	63.3 ± 14.6	62.8 ± 18.2	58.1 ± 18.6	60.3 ± 18
SARS-CoV-2 vaccination	–	–	21 (13%)	30 (39.5%)	76 (19.3%)
Risk factors for severe COVID-19					
Age > 65 years	29 (59.2%)	21 (48.8%)	84 (51.9%)	30 (39.5%)	186 (47.2%)
BMI > 25 kg/m^2^	8 (16.3%)	12 (27.9%)	38 (23.5%)	17 (22.4%)	92 (23.4%)
Hypertension	26 (53.1%)	18 (41.9%)	68 (42%)	30 (39.5%)	164 (41.6%)
Diabetes mellitus	13 (26.5%)	5 (11.6%)	29 (17.9%)	19 (25%)	80 (20.3%)
CKD	9 (18.4%)	4 (9.3%)	18 (11.1%)	11 (14.5%)	40 (10.2%)
Heart disease	19 (38.8%)	11 (25.6%)	44 (27.2%)	19 (25%)	98 (24.9%)
COPD	5 (10.2%)	1 (2.3%)	13 (8%)	6 (7.9%)	24 (6.1%)
Neurological disease	7 (14.3%)	6 (14%)	19 (11.7%)	8 (10.5%)	38 (9.6%)
Number of risk factors					
0	0 (0%)	0 (0%)	0 (0%)	0 (0%)	47 (11.9%)
1	2 (4.1%)	6 (14%)	18 (11.1%)	0 (0%)	95 (24.1%)
2	9 (18.4%)	8 (18.6%)	33 (20.4%)	28 (36.8%)	80 (20.3%)
3	9 (18.4%)	6 (14%)	29 (17.9%)	16 (21.1%)	73 (18.5%)
4	15 (30.6%)	12 (27.9%)	41 (25.3%)	8 (10.5%)	42 (10.7%)
5	7 (14.3%)	6 (14%)	17 (10.5%)	9 (11.8%)	35 (8.9%)
6	5 (10.2%)	3 (7%)	15 (9.3%)	11 (14.5%)	16 (4.1%)
7	2 (4.1%)	2 (4.7%)	8 (4.9%)	1 (1.3%)	6 (1.5%)
WHO ordinal scale					
1	–	–	6 (3.7%)	4 (5.3%)	11 (2.8%)
2	–	–	2 (1.2%)	5 (6.6%)	10 (2.5%)
3	–	–	41 (25.3%)	44 (57.9%)	131 (33.2%)
4	18 (36.7%)	37 (86%)	57 (35.2%)	20 (26.3%)	134 (34%)
5	11 (22.4%)	2 (4.7%)	19 (11.7%)	0 (0%)	42 (10.7%)
6	2 (4.1%)	1 (2.3%)	3 (1.9%)	0 (0%)	9 (2.3%)
7	2 (4.1%)	0 (0%)	2 (1.2%)	0 (0%)	3 (0.8%)
8	16 (32.7%)	3 (7%)	32 (19.8%)	3 (3.9%)	54 (13.7%)

*SD* standard deviation, *BMI* body mass index, *CKD* chronic kidney disease, *COPD* chronic obstructive pulmonary disease, *IQR* interquartile range, *ICU* intensive care unit

The results of the descriptive statistical analysis revealed a notable association between the number of risk factors and a worse WHO ordinal scale score, particularly with respect to the escalation of treatment to oxygen therapy and mortality rates (Fig. 1). Of note, age > 65 years was found to be an outstanding predictor of worse WHO ordinal scale outcome, as demonstrated by the substantial increases observed in rates of oxygen therapy and death (Fig. 2).

**Fig. 1:**
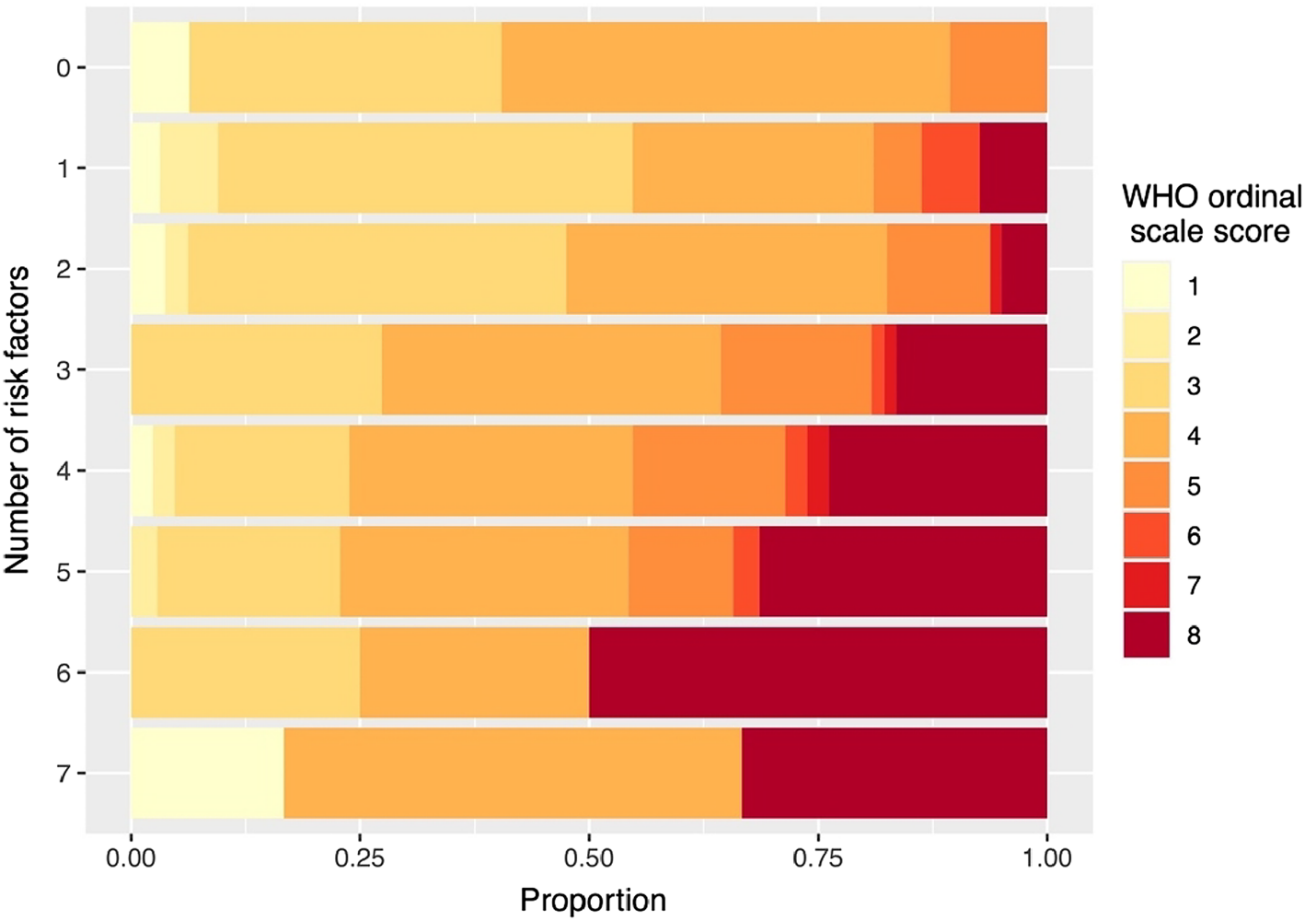
Stacked bar chart depicting the relationship between the number of risk factors and the proportion of WHO ordinal scale outcomes, with darker shades indicating worse outcomes

**Fig. 2:**
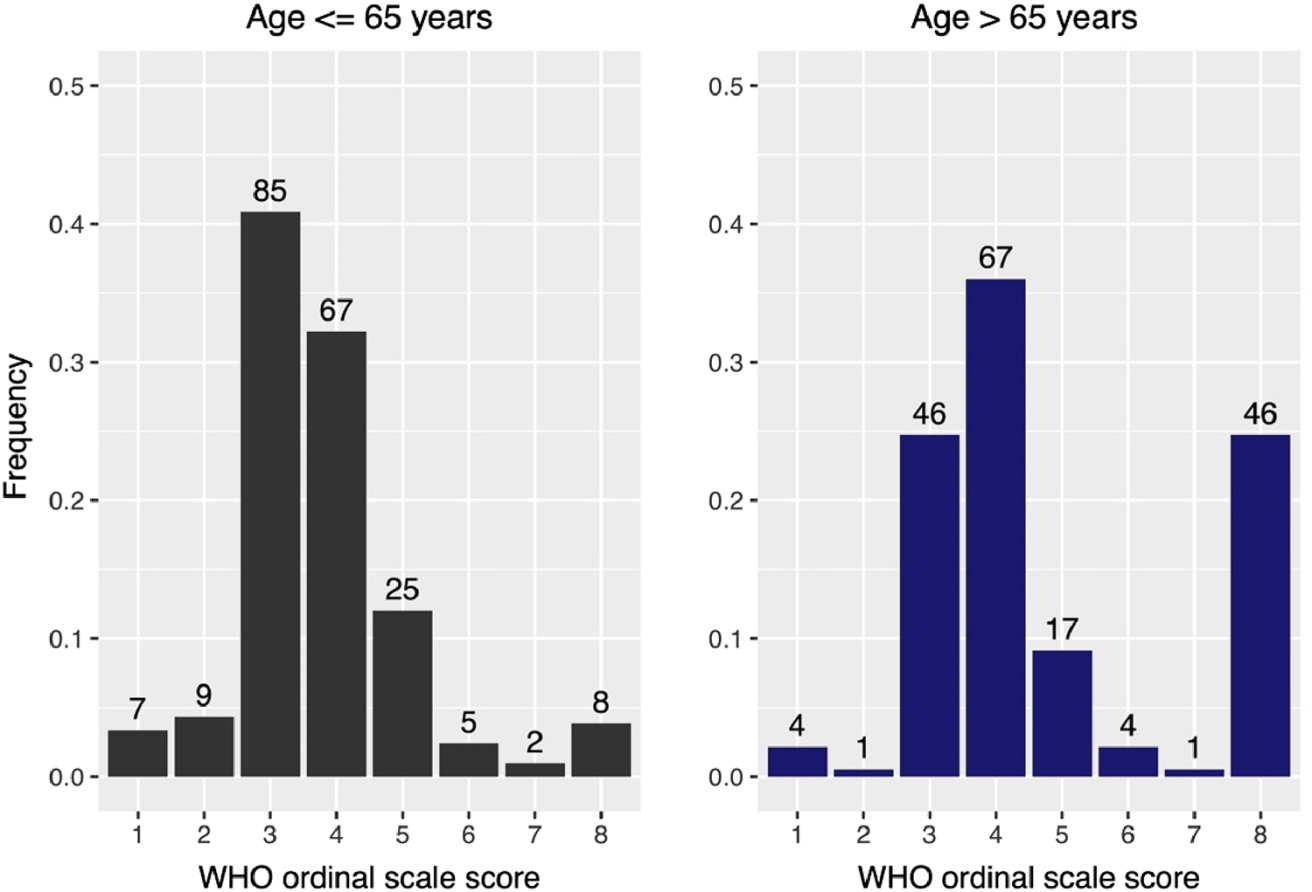
Distribution of WHO ordinal scale scores in patients under and over the age of 65 years

Our findings indicate that RDV treatment in patients with advanced COVID-19 (median time of 7 days between diagnosis and treatment initiation; IQR 4–10 days) and need for oxygen support was associated with a significantly lower probability of worse WHO ordinal scale outcomes compared to untreated controls (OR 0.12, 95% CI 0.05–0.29, p < 0.001) (Fig. 3). We additionally calculated cumulative incidence functions: a significantly reduced time until hospital discharge for patients treated with RDV was demonstrated based on the 95% CI (Fig. 4). An association of treatment with RDV and a shortened time until testing negative for COVID-19 could not be shown (Fig. 5).

**Fig. 3:**
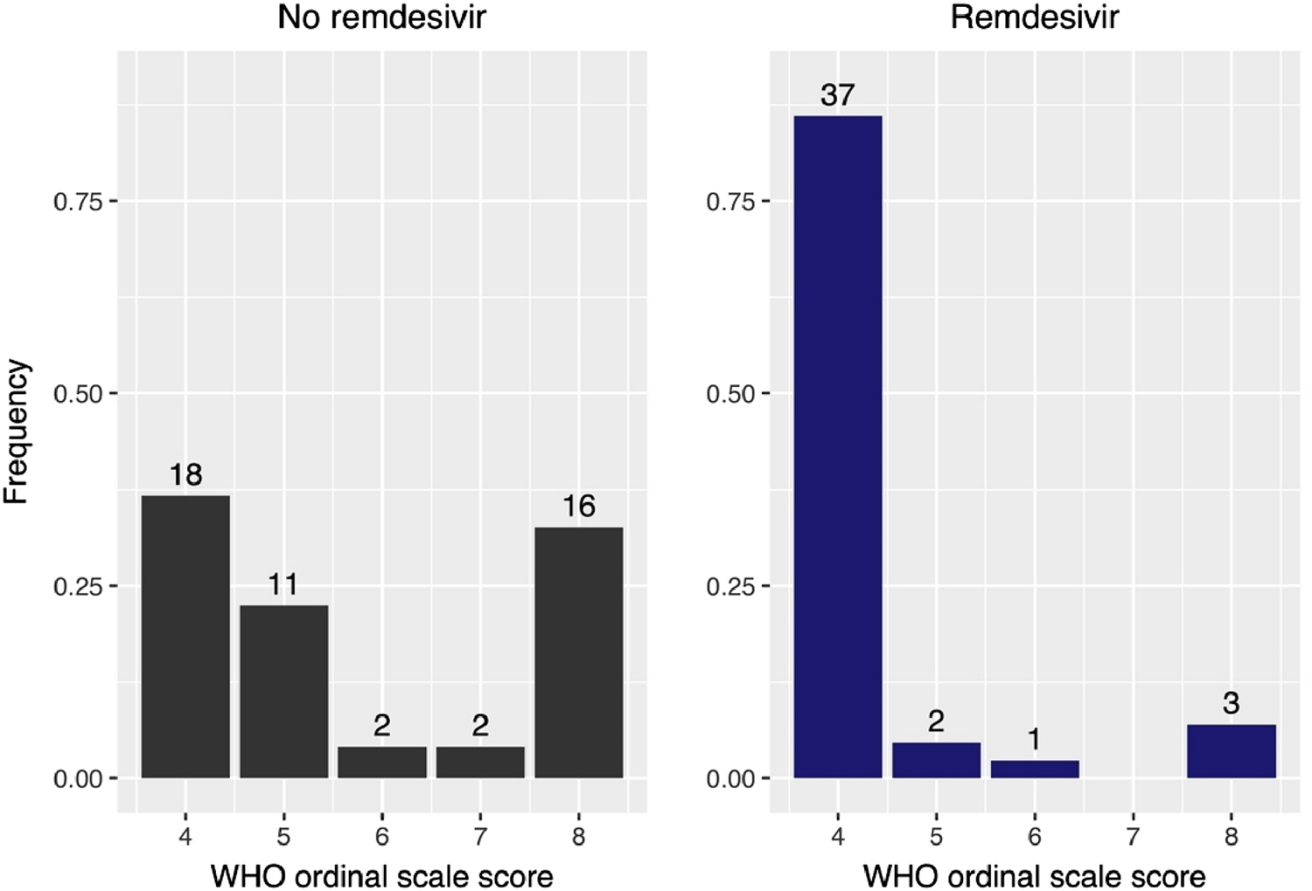
Distribution of WHO ordinal scale scores for patients treated with or without remdesivir (RDV)

**Fig. 4:**
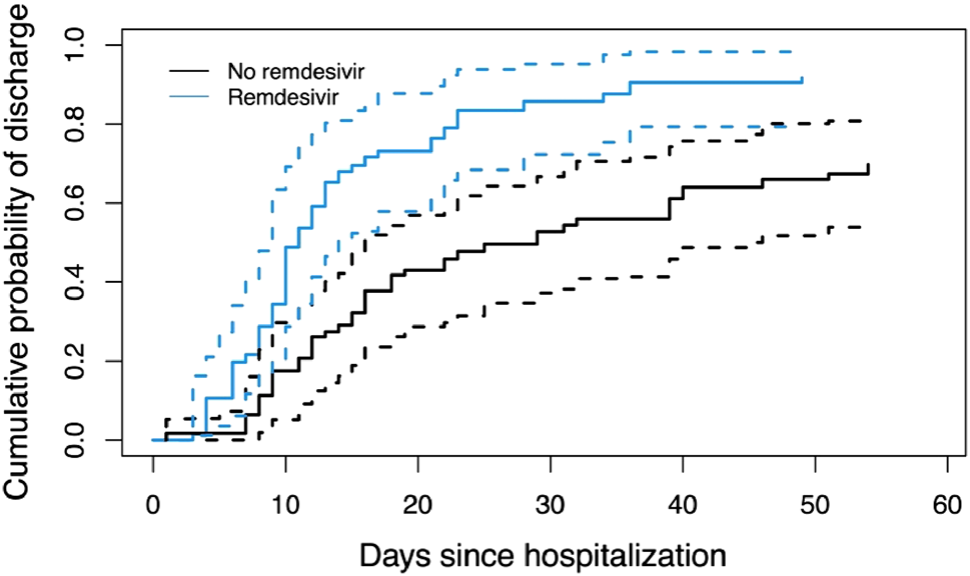
Cumulative incidence function estimates based on the inverse probability weighted data for duration of hospitalization in days displaying the cumulative probability of discharge for patients hospitalized with COVID-19 (RDV vs. no RDV). Both curves are accompanied by dotted lines indicating the 95% confidence interval

**Fig. 5:**
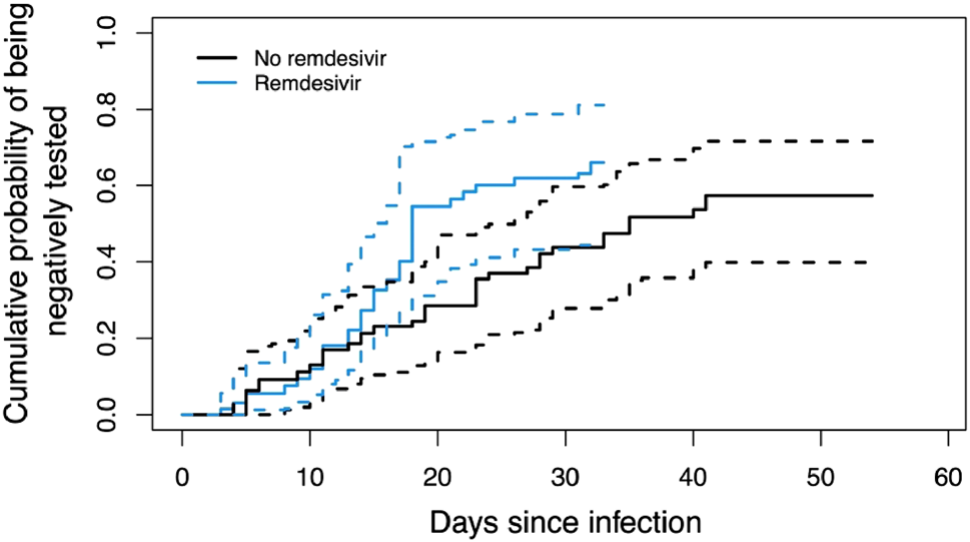
Cumulative incidence function estimate based on the weighted data showing the probability of testing negative for COVID-19 over time since a positive test result (RDV vs. no RDV). Both curves are accompanied by dotted lines indicating the 95% confidence interval

Finally, early treatment initiation with CVIV within the first five days after diagnosis of COVID-19 in patients at high risk for a severe course was found to prevent the development of a WHO ordinal scale > 3 (OR 0.25, 95% CI 0.12–0.55, p < 0.001), i.e., requiring oxygen (Fig. 6).

**Fig. 6:**
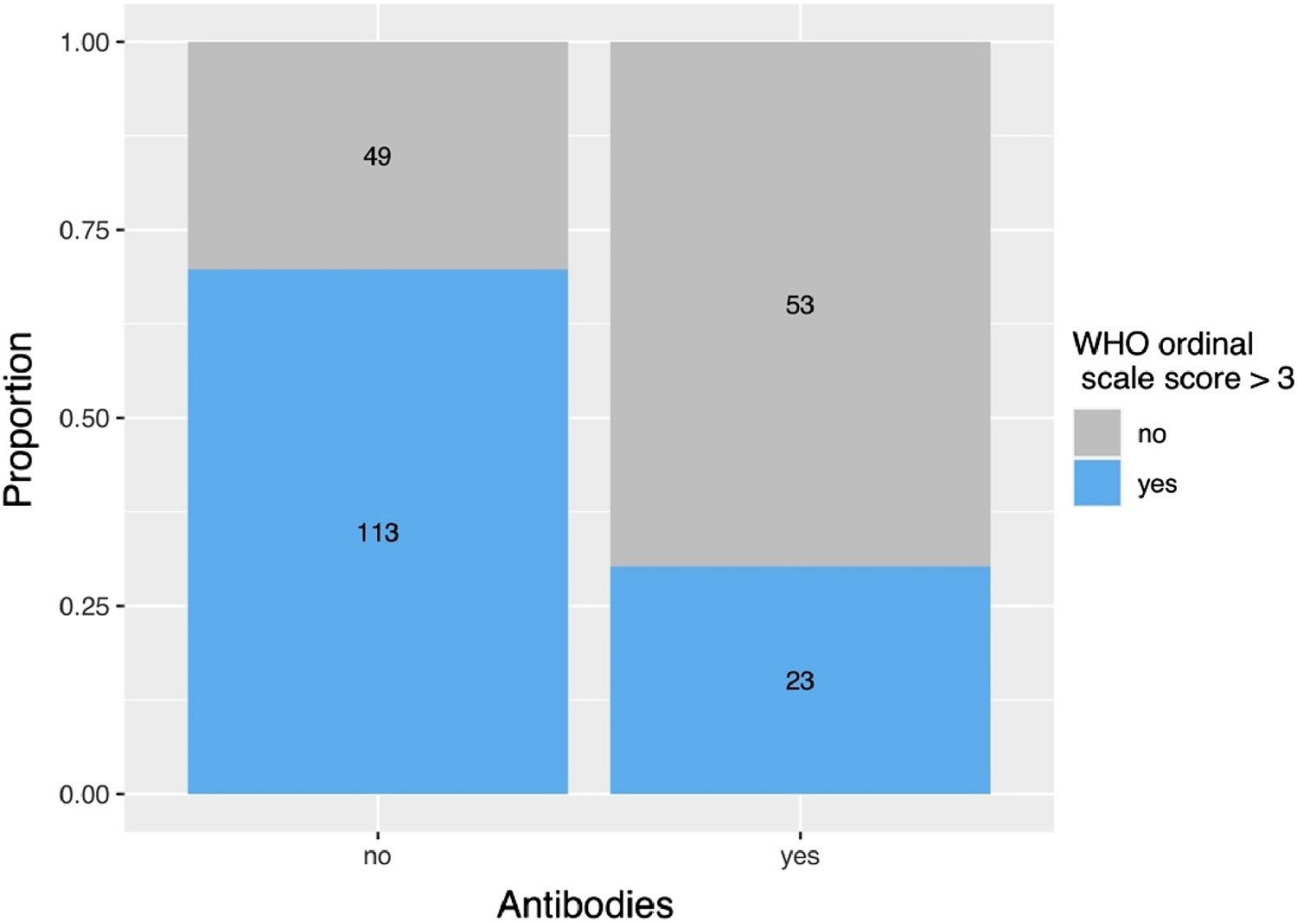
Bar chart depicting the distribution of COVID-19 patients who received antibody treatment (no/yes) in relation to their WHO ordinal scale outcome

Our study adds clinical data to the growing evidence supporting the use of RDV in patients with advanced COVID-19—especially in unvaccinated patients. Using the WHO ordinal scale, we found that clinical course was better in patients in need for oxygen support who received RDV thereby supporting the data of Pilgram et al. [1].

RDV was used in the late phase of infection in patients requiring oxygen—and thereby in part outside the current indication. High-risk patients who have missed early therapy with DAAs and/or mABs can therefore benefit from the use of RDV in the event of clinical deterioration.

However, the decisive use of mABs such as CVIV in the early phase of infection can effectively prevent such events. mABs have strongly been recommended due to their efficacy and lack of severe side effects [2]. Yet, the emergence of new viral variants complicates the use of mABs, as efficacy dropped dramatically with mutations at the SARS-CoV-2 spike protein. Currently, all available mABs have rapidly experienced a loss of efficacy due to viral evolution. Therefore, the determination of the underlying SARS-CoV-2 variant plays a special role for individualised therapy decisions. The future use of mABs depends on newly developed mABs targeting evolving virus strains.

In comparison, RDV as a nucleotide-like antiviral drug against the SARS-CoV-2 RNA polymerase does not seem to have been affected by mutations in the past. RDV should therefore been used in the early and/or in the late phase of infection outside the current indication, especially in immunological naïve patients with regard to SARS-CoV-2 who are at high risk for severe COVID-19.

The WHO ordinal scale is a simple and easy-to-use tool that measures the disease course in clinical status based on a 9-point ordinal scale. The scale ranges from no clinical or virological evidence of infection (0) to death (8) and includes several intermediate categories that reflect different degrees of clinical deterioration. Significant steps in the progression of the disease include the transition from mild (WHO ordinal scale 1–3) to moderate disease, requiring supportive oxygen therapy (WHO ordinal scale 4). Additionally, the progression to severe disease involves the use of mechanical ventilation, such as non-invasive and/or invasive ventilation, and falls within the WHO ordinal scale 5–7 range [3].

The use of WHO ordinal scale has several benefits, including standardization of outcome measurement, ease of implementation, and applicability across different treatment modalities. Thus, the WHO ordinal scale can simplify the handling of COVID-19 for specialists from other clinical disciplines than infectious disease. Consequently, we think that the use of the WHO ordinal scale remains useful both as a tool for clinical decision-making and for therapy evaluation.

It should be emphasized that an even more reliable classification into risk groups could be made with the implementation of inflammation markers resulting in a combined ordinal scale. In this case, each of the three previously mentioned groups would be divided into low risk and high risk [3]. Nevertheless, we see clear advantages using the simple WHO ordinal scale based only on clinical data, especially for general practitioners and specialists from other clinical disciplines than infectious disease—especially for reasons of time in a fast-paced everyday life.

While there are many studies supporting the use of PCR results as outcome measures, SARS-CoV-2 viral load has limited utility in evaluating treatment outcomes in COVID-19 patients especially due to prolonged viral shedding in high-risk patients. It should only be used as a secondary readout. Hospital discharge should not be reliant on negative PCR results since SARS-CoV-2 viral shedding kinetics make those results unreliable [5].

Patients with underlying oncological/haematological diseases and/or B-cell-depleting therapies are particularly at risk of severe courses of COVID-19. Only a few of these patients (n = 9) were included in our analysis on RDV, which limits our data. Nevertheless, especially SARS-CoV-2 immunologically naïve patients are still at risk for severe courses of COVID-19. If they have risk factors for COVID-19, such as age over 65 years, hypertension, obesity, diabetes, chronic kidney disease, heart disease, chronic obstructive pulmonary disease, and/or neurological disease (which were considered for our propensity scores), they should also be treated.

In conclusion, our data support the findings of the LEOSS registry regarding the efficacy of RDV in improving clinical outcomes in patients with advanced COVID-19 [1]. Our study provides important insights into the use of RDV in advanced COVID-19, clinical decision-making and the evaluation of COVID-19 treatments.

## Data Availability

The data are available on request.
